# Ozanimod-mediated remission in experimental autoimmune encephalomyelitis is associated with enhanced activity of CNS CD27^low/-^ NK cell subset

**DOI:** 10.3389/fimmu.2024.1230735

**Published:** 2024-03-12

**Authors:** Doua Kamyan, Maya Hassane, Alanood Alnaqbi, Abdul-Kader Souid, Zakeya Al Rasbi, Abeer Al Tahrawi, Mariam Al Shamsi

**Affiliations:** ^1^ Department of Medical Microbiology and Immunology, College of Medicine and Health Sciences, United Arab Emirates (UAE) University, Al Ain, Abu Dhabi, United Arab Emirates; ^2^ Department of Pediatrics, College of Medicine and Health Sciences, United Arab Emirates (UAE) University, Al Ain, Abu Dhabi, United Arab Emirates; ^3^ Department of Pathology, College of Medicine and Health Sciences, United Arab Emirates (UAE) University, Al Ain, Abu Dhabi, United Arab Emirates

**Keywords:** multiple sclerosis, experimental autoimmune encephalomyelitis, natural killer cells, ozanimod, sphingosine-1-phosphate, sphingosine-1-phosphate receptor

## Abstract

**Background:**

Ozanimod (RPC1063) is an immunomodulator that has been recently approved by the FDA (2020) for the treatment of relapsing-remitting multiple sclerosis (RRMS). It is a selective agonist of the sphingosine-1-phophate receptors 1 and 5, expressed on naïve and central memory T and B cells, as well as natural killer (NK) cells, and is involved in lymphocyte trafficking. Oral administration of ozanimod was reported to result in rapid and reversible reduction in circulating lymphocytes in multiple sclerosis (MS) patients, however, only minimal effect on NK cells was observed. In this study, we sought to investigate the effect of ozanimod on NK cells and assess whether they play any role in ozanimod-induced remission in experimental autoimmune encephalomyelitis (EAE), the animal model of MS.

**Methods:**

Active EAE induction was done in C57BL/6 female mice, followed by daily oral treatment with ozanimod (0.6mg/kg) starting at disease onset (score 1). Flow cytometry of blood and CNS was performed 24 hours after the last oral dose of ozanimod treatment in diseased mice. Histological analysis of lumbar spinal cord was performed for evaluating the level of inflammation and demyelination. Depletion of peripheral NK cells was done using anti-NK1.1 mouse antibody (mAb) at day 5 post-EAE induction.

**Results:**

Ozanimod was effective in reducing the clinical severity of EAE and reducing the percentage of autoreactive CD4^+^ and CD8^+^ T cells along with significant inhibition of lymphocyte infiltration into the spinal cord, accompanied by reversed demyelination. Furthermore, ozanimod treatment resulted in a significant increase in the frequency of total NK cells in the blood and CNS along with upregulation of the activating receptor NKG2D on CD27^low/-^ NK cell subset in the CNS. The effectiveness of ozanimod treatment in inhibiting the progression of the disease was reduced when NK cells were depleted using anti-NK1.1 mAb.

**Conclusion:**

The current study demonstrated that ozanimod treatment significantly improved clinical symptoms in EAE mice. Ozanimod and anti-NK1.1 mAb appear to function in opposition to one another. Collectively, our data suggest that ozanimod-mediated remission is associated with an increased percentage of total NK cells and CD27^low/-^ NK cells expressing the activating receptor, NKG2D in the CNS.

## 1 Introduction

Multiple sclerosis (MS) is a chronic inflammatory autoimmune neurodegenerative disease of the central nervous system (CNS) which leads, in many cases, to irreversible disability. Globally, approximately 2.8 million people live with MS, with onset between 20 and 40 years of age, and women being two times more likely to develop the disease than men (1–3). Relapsing-remitting (RR) MS is the most common subtype of MS and constitutes 85–90% of young patients. RRMS manifests as unpredictable and acute attacks (relapses) causing deterioration of CNS functions over time, followed by partial or complete recovery (remissions) (4, 5). The disease onset is thought to be initiated by the activation of autoreactive naive CD4^+^ T cells against components of the CNS in the periphery and subsequent infiltration of the activated lymphocytes into the CNS through disrupted blood brain barrier (BBB). This is also thought to be followed by expansion of local and infiltrating peripheral leukocytes and their contribution to the inflammatory milieu within the CNS, resulting in demyelination and axonal damage (6, 7). MS has been studied using different animal models, with experimental autoimmune encephalomyelitis (EAE) being the most commonly employed murine model to understand MS pathogenesis and possible therapeutic targets (8).

The sphingolipid sphingosine-1-phosphate (S1P) is known for its important function in regulating immunity through affecting trafficking, differentiation, and survival of immune cells. Expression of S1PRs on lymphocytes is essential for regulation of their homing and migration through the primary and secondary lymphoid organs, as well as inflamed sites (9). There are different S1PRs expressed by lymphocytes such as T cells (S1PR1 and S1PR4), B cells (S1PR1, S1PR2, S1PR3, and S1PR4), while NK cells express low levels of S1PR1 and higher levels of S1PR5 (10). In addition, S1PRs are expressed at high levels on CNS cells, including neurons, oligodendrocytes, astrocytes, and microglia, enabling S1PRs agonists to exert different effects within the CNS (11). S1P agonists such as fingolimod and the recently FDA approved drug, ozanimod, have been reported to result in significant amelioration in the severity of MS and EAE, mainly attributed to interfering with infiltration of activated autoreactive T lymphocytes expressing S1PR1 into the CNS (12, 13). Even though the S1P modulators are agonists of the receptor, binding to the S1PRs results in internalization of the receptor-modulator complex into the lymphocytes, preventing their egress from the lymph nodes and therefore acting as an effective antagonist (14, 15). While fingolimod (FTY720), the first approved sphingosine-1-phosphate receptor (S1PR) modulator for treatment of MS, targets cells expressing S1P receptors 1, 3, 4 and 5 (12), ozanimod is more selective, only targeting S1PR1 and S1PR5 (16). Due to its selectivity, ozanimod offers a better risk-benefit profile compared to fingolimod, making treatment with ozanimod safer and more effective (13).

It is noteworthy to mention that treatment with S1PR agonists (ozanimod) also is associated with a significant decrease of circulating lymphocytes counts in a dose-dependent manner. However, rapid lymphocytes count recovery was observed after discontinuation of the treatment because of its short half-life of 19 hours, allowing for once-daily dosing (9, 13).

NK cells are large granulated innate lymphoid cells (ILCs), best known for their ability to identify and induce apoptosis in virus-infected cells and cancer cells (17). Majority of mature NK cells in humans or mice consistently display NK group 2, member D (NKG2D), CD161, NK-cell protein 46 (NKp46), and CD122. Nonetheless, variations in the expression of Ly49 family members, CD127, CD27, and KLRG1 (killer-cell lectin-like receptor subfamily G, member 1) in mice, as well as KIRs (killer-cell immunoglobulin-like receptors), CD56, and CD16 in humans, indicate diversity within the mature NK-cell population (18). Based on the expression of CD56 marker, human NK cells are subdivided into two major cell subsets, CD56^dim^ and CD56^bright^. The CD56^dim^ NK cell subset represents the mature cytotoxic subset that makes up to 90% of the total circulating NK cells. In contrast, the immature CD56^bright^ NK cell subset is known for its efficiency in cytokines production and represents around 10% of the NK cell pool in the periphery. NK cells in C57BL/6 mice express NK1.1 and CD27, therefore, they can be subdivided into CD27^high^ and CD27^low/-^, representing the equivalent of human CD56^bright^ and CD56^dim^ NK cell subsets, respectively (18–22). It has been suggested that the two subsets of NK cells, CD27^high^ and CD27^low/-^, play different roles in the disease progression and at different stages (23). It is noteworthy that the number of circulating CD56^bright^ NK cells in MS patients was suggested as an indicator for therapeutic efficacy since in a number of immunomodulatory treatments (e.g. daclizumab and dimethyl fumarate), the level of CD56^bright^ NK cells was found to be increased. This increase was also accompanied with improved cytotoxic activity towards autoreactive CD4^+^ T cells and reduced disease severity (24–28). On the contrary, treatment of MS patients with fingolimod was reported to be associated with a decrease in the number of circulating CD56^bright^ NK cells (29, 30), and a significant increase in an aged and less functional phenotype (CD56^dim^ CD94^low^ mature and less functional NK cells) (28).

It has not yet been studied how treatment with the selective S1PR modulator, ozanimod, affects NK cells and their subsets. Aside from ozanimod’s reported effects on other lymphocytes (CD4^+^, CD8^+^ T cells, and B cells), we are also interested in whether it is associated with an expansion in a specific NK cell subset.

In the present study, we demonstrated that oral administration of ozanimod is effective in reducing the number of relapses and suppressing the disease progression in EAE C57BL/6 mice through markedly decreasing number of infiltrating immune cells and reversing demyelination. Further, we investigated the effect of ozanimod on NK cells and their activation status. Our findings suggest that ozanimod increases the frequency of NK cells in the blood and CNS and enhances activation of CD27^low/-^ NK cell subset in the CNS, which might be associated with improvements in motor function and overall EAE disability.

## 2 Materials and methods

### 2.1 Mice

A total of 67 C57BL/6 female mice were used at 10 to13 weeks of age. Mice were housed at 22°C with 60% humidity and 12-h light-dark cycles. Rodent chow and filtered water were provided. Wet rodent chow and filtered water were made always accessible to mice with all disease scores.

### 2.2 Active induction of relapsing-remitting EAE

EAE was induced in C57BL/6 female mice by injecting them with 200µg of MOG_35-55_ (myelin oligodendrocyte glycoprotein 35-55; AnaSpec, USA) peptide emulsified in incomplete Freund’s adjuvant (IFA) (Sigma, St Louis, MO, USA) containing 3mg/ml inactivated *Mycobacterium tuberculosis* H37Ra (Becton, Dickinson and company, Sparks, MD 21152 USA) subcutaneously at the back on day 0, followed by intraperitoneal injections of 500ng of pertussis toxin (Sigma, St Louis, MO, USA) on days 0 and 2. Control mice received a subcutaneous injection of IFA at the back. Mice were weighed daily and monitored for disease progression using the standard clinical scoring system of paralytic EAE: score 0, no disease; 1, flaccid tail; 2, hind limbs weakness and wobbly gait; 3, paralysis of hind limbs with residual mobility in both legs; 4, both hind legs completely paralyzed and partial front limb paralysis; 5, moribund or death (7). Wet food pellets were provided inside the cage for mice with advanced disease scores (score 3 and above).

### 2.3 Ozanimod treatment

Following disease induction, mice were randomly enrolled into different groups (7-12 mice per group). At the first sign of disease, flaccid tail, diseased mice received daily administration of ozanimod (OZ) (RPC1063) (SelleckChem) at 0.6mg/kg diluted in 5% DMSO, 5% Tween-20 and 90% 0.1N HCl by oral gavage (15) for up to 21 days. The control group was orally treated with the diluent of ozanimod (5% DMSO, 5% Tween-20 and 90% 0.1N HCl).

### 2.4 Histological assessment of demyelination and inflammation in spinal cords

Mice were euthanized by intraperitoneal injection of ketamine (87.5 mg/kg) and xylazine (12.5mg/kg) (Ilium, Smithfield, NSW, Australia) followed by intracardiac perfusion with PBS. Spinal cord was removed, and the lumbar region (L1-L5) was excised and fixed in 10% formalin overnight at room temperature (RT). A series of paraffin-embedded 7 and 10μm thick lumbar spinal cord tissue sections were cut and deparaffinized in xylene followed by hydration in gradual decreasing concentrations of ethanol. H&E staining was performed on the 7μm tissue sections to assess mononuclear cells infiltration using H&E staining kit (IHC World, USA) following the manufacturer’s protocol. Spinal cord sections of 10μm were stained with Luxol fast blue (LFB) to assess the level of demyelination in diseased mice. Staining was performed using LFB staining kit (IHC World, USA) and following manufacturer’s guidance.

Interpretational analysis of the H&E and LFB stained sections was done in a blinded manner. Inflammatory score was evaluated using the following reporting criteria: 0, no inflammation; 1, cellular infiltration only in the perivascular areas and meninges; 2, mild cellular infiltration (less than one-third part of the white matter is infiltrated with inflammatory cells); 3, moderate cellular infiltration (more than one-third part of the white matter is infiltrated with inflammatory cells); and 4, infiltration of inflammatory cells is observed in the entire white matter (7). The assessment of demyelination was done by estimating the percentage of the demyelinated area. Cresyl violet stained neurons with violet and LFB solution stained myelin with blue-green color, loss of blue-green stain in the white matter is an indication of demyelination.

### 2.5 Isolation of mononuclear cells from the CNS

Brain and spinal cord were removed from euthanized and PBS perfused mice and were cut into small pieces that were mechanically dissociated by syringe plunger and filtered through a 70μm cell strainer. Cell suspension was transferred into a 50ml conical tube, topped with PBS and centrifuged at 1400 rpm for 10 min at 4°C. The cell pellet was resuspended in 20ml PBS + 30% Percoll (GE Healthcare) and was overlaid with 10ml PBS + 70% Percoll and centrifuged at 2800 rpm for 30 min at RT without brake. The myelin on top of the tube was removed and mononuclear cells in the 30–70% Percoll interface were collected, transferred to a 50ml conical tube and washed twice with PBS. Cells were resuspended in 1-2ml of FACS buffer (PBS, 0.5% BSA, and 0.1% NaN3), centrifuged and incubated with FACS antibodies for 25 min at 4°C protected from light.

### 2.6 Isolation of circulating peripheral blood mononuclear cells

Cardiac blood was collected in an EDTA tube. 1ml of red blood cell lysis buffer was added per 100μl of blood and was incubated for 5 min at RT. Reaction was stopped by adding 20ml PBS followed by centrifugation at 1400 rpm for 10 min at 4°C. Cell pellet was washed once with PBS then resuspended in 1-2ml of FACS buffer. Cells were then incubated with the antibodies for 25 min at 4°C protected from light.

### 2.7 Reagents and antibodies for flow cytometry analysis

Antibodies against mouse CD45 (BV605-conjugated, clone 30-F11), CD4 (BV785-conjugated, clone RM4-5), CD8a (APCCY7-conjugated, clone 53-6.7), CD3 (BV421-conjugated, clone 17A2), NK1.1 (FITC-conjugated, clone PK136), CD27 (BV510-conjugated, clone LG.3A10), NKG2A (PE-conjugated, clone 16A11), NKG2D (APC-conjugated, clone CX5) and appropriate isotype controls were purchased from BioLegend (SanDiego, CA, USA). Gating strategies used in this study are presented in **Supplementary Figure 1**. Data were collected using BD FACSCelesta™ Cell Analyzer from BD Biosciences (Germany) and analyzed using FlowJo-v 10.8.1 (FlowJo, LLC, Ashland, OR, USA). Anti-NK1.1 mAb (clone PK136) and its appropriate isotype control were purchased from BioXcell, USA.

### 2.8 *In vivo* depletion of NK cells

*In vivo* depletion of NK cells was achieved through i.p. administration of 100µg of anti-NK1.1 mAb or IgG2a isotype control (BioXcell, USA) at day 5 post-EAE induction. Injection was repeated with lowered dose (50µg) of anti-NK1.1 mAb or IgG2a isotype control every 5 days until the end of the experiment (21 days after disease onset) (29).

### 2.9 Statistical analysis

All statistical analysis were performed using GraphPad Prism V10. Briefly, all data were tested for normality using Shapiro-Wilk test. For non-normally distributed data, a non-parametric test (Mann-Whitney U test) was used. Statistical significance was defined as *p*- values ≤ 0.05. Data are represented as mean ± SD.

### 2.10 Ethical approval

The study was approved by the UAE University Animal Research Ethics Committee (Ref. ERA_2021_7326).

## 3 Results

### 3.1 Ozanimod treatment halted EAE progression and reduced body weight loss in diseased mice

**Figure 1:**
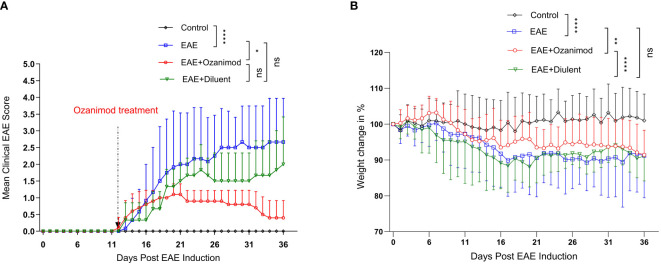
Therapeutic effect of ozanimod (RPC 1063) on the clinical score and body weight of EAE mice. **(A)** The clinical scores show a statistical difference between ozanimod-treated and untreated diseased mice. **(B)** The weight changes indicate a significant inhibition on further body weight loss in ozanimod-treated EAE mice compared to untreated mice. Data pooled from two independent experiments are shown (8-12 mice per group). Clinical scores and body weights (Percentage of the weight divided by starting weight) are shown. Data are represented as mean ± SD using Mann-Whitney *U* test. ns P > 0.05, * P ≤ 0.05, ** P ≤ 0.01, **** P ≤ 0.0001.

RR-EAE was successfully induced in female C57BL/6 mice through s.c. administration of MOG_35-55_ peptide emulsified in IFA. Average time for EAE onset in all diseased groups was between days 12 and 15, while peak was between days 18 and 24 (scores 2 and 3) (**Figure 1A**) in comparison to control mice which maintained a clinical score of 0 throughout the study duration (36 days).

Ozanimod treatment was initiated at disease onset (score 1, limp tail), and carried on for 21 days. The therapeutic potential of ozanimod in halting EAE progression started to become evident at day 22 (mean maximum clinical score MMCS 0.9 ± 0.3) and continued throughout the treatment, by the end of which 60% of the mice were at scores 0 and 40% were at score 1. Disease severity in ozanimod-treated mice was significantly reduced compared to their diseased untreated counterparts (P=0.015). Furthermore, diseased mice from both EAE and EAE+diluent groups experienced relapses starting from day 25, whereas no relapses were observed in the ozanimod-treated group, indicating the effectiveness of ozanimod in preventing relapses (**Figure 1A**). Individual clinical scores of mice (**Supplementary Figure 2**) further highlight these findings.

Treatment with ozanimod significantly prevented further weight loss in comparison to the control diseased groups whether treated or not with ozanimod’s diluent (P=0.0001 and 0.001, respectively) (**Figure 1B**). Diseased mice groups reported significant reduction in the body weight compared to the control group (P<0.0001), with a reduction of 8.8%, and 8.9% in EAE and EAE+diluent groups, respectively, compared to EAE+ozanimod group (7.5%) (**Figure 1B**). Altogether, these data indicate that treatment with ozanimod significantly improved disease outcome by reducing disease severity and weight loss.

### 3.2 Ozanimod treatment reduced mononuclear cell infiltration and level of demyelination in the spinal cords of EAE mice

**Figure 2:**
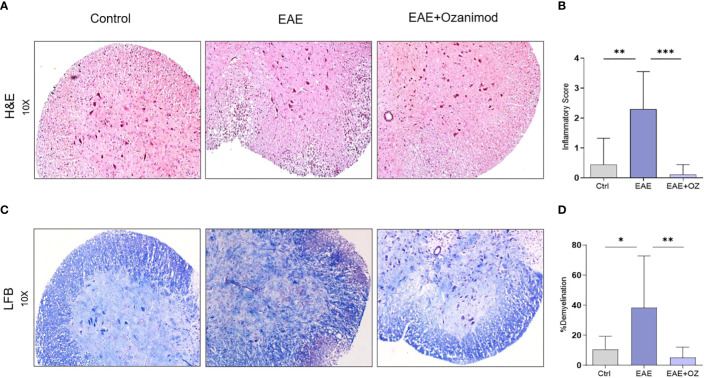
Histological sections of the lumbar region of the spinal cord obtained from control, EAE, and ozanimod-treated EAE mice. **(A, B)** H&E staining, performed to detect mononuclear cell infiltration level, shows a significant reduction in the given inflammatory score in ozanimod-treated EAE mice compared to untreated mice. **(C, D)** LFB staining, performed to detect the level of demyelination, indicates decreased demyelination in the white matter in ozanimod-treated EAE mice compared to those untreated as shown by reduced loss in the blue staining. Histological scores and percentages of demyelination are shown. Data are represented as mean ± SD using Mann-Whitney *U* test. * P ≤ 0.05, ** P ≤ 0.01, *** P ≤ 0.001.

Assessing inflammation (infiltration of mononuclear cells) and demyelination as pathological signs of the disease in the spinal cord sections of control, EAE and EAE+ozanimod mice groups was achieved by analyzing histology sections stained with H&E and LFB. H&E staining showed significant increase in the infiltration mononuclear cells in the white matter and perivascular region of the spinal cords of EAE mice group (mean inflammatory score (MIS) =2.3) compared to control (MIS=0.4) and ozanimod-treated (MIS=0.1) groups (**Figures 2A, B**). LFB staining revealed a significant increase in the percentage of demyelination, as exhibited by the loss of LFB staining and vacuolization, in the EAE group (38.3%) compared to their control counterparts (10.5%). This percentage was significantly decreased in ozanimod-treated mice (5%) (**Figures 2C, D**). These results indicate effectiveness of ozanimod in reducing inflammation, presented by mononuclear cell infiltration, and demyelination in the spinal cords of diseased mice.

### 3.3 Ozanimod reduced the frequency of circulating CD4^+^ and CD8^+^ T cells in EAE mice

**Figure 3:**
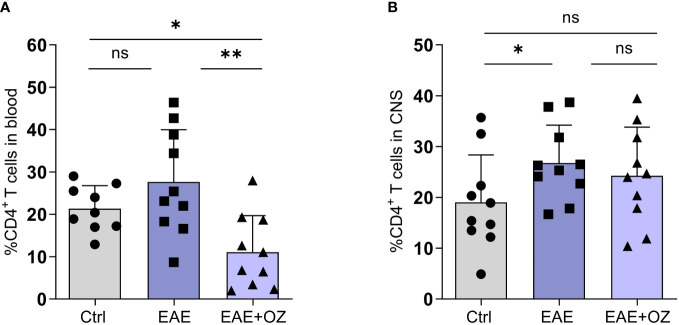
Frequencies of CD4^+^ T cells in the blood circulation and CNS of EAE mice. **(A)** Significant decrease in the frequency of circulating CD4^+^ T cells in ozanimod-treated EAE mice compared to those untreated. **(B)** No significant changes were detected in the frequency of CNS infiltrating CD4^+^ T cells in ozanimod-treated EAE mice compared to those untreated. Representative data from two independent experiments are shown (9-10 mice per group). Data are represented as mean ± SD using Mann-Whitney *U* test. ns P > 0.05, * P ≤ 0.05, ** P ≤ 0.01.

Changes in the frequency of CD4^+^ and CD8^+^ T cells in the blood and CNS were studied as part of confirming the therapeutic potential of ozanimod treatment. Flow cytometry analysis of blood revealed a significant reduction in the frequency of CD4^+^ T cells in ozanimod-treated EAE mice compared to their untreated counterparts (**Figure 3A**). Frequency of CD4^+^ T cells infiltrating the CNS increased significantly in EAE mice compared to control group (P=0.03) but remained unchanged following ozanimod treatment (**Figure 3B**). Additionally, there was a significant decrease in the frequency of CD8^+^ T cells in the blood and CNS in ozanimod-treated EAE mice as opposed to their untreated counterparts (**Supplementary Figure 3**). These results suggest that circulating CD4^+^ and CD8^+^ T cells are significantly affected by the immunomodulatory drug ozanimod.

### 3.4 Ozanimod treatment increased the frequency of circulating and CNS NK cells

**Figure 4:**
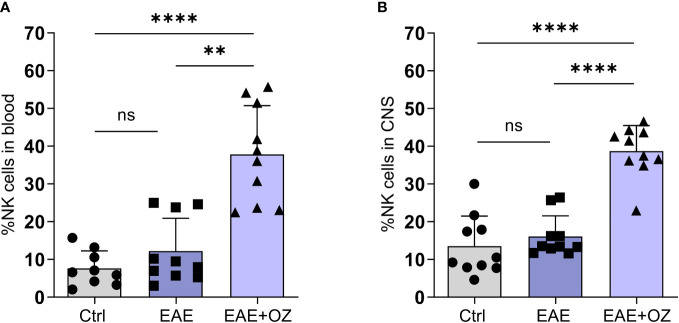
Frequencies of NK cells in the blood circulation and CNS of EAE mice. Treatment with ozanimod resulted in increased frequency of total **(A)** circulating NK cells and **(B)** CNS NK cells in EAE mice compared to untreated mice. Representative data from two independent experiments are shown (9-10 mice per group). Data are represented as mean ± SD using Mann-Whitney *U* test. ns P > 0.05, ** P ≤ 0.01, **** P ≤ 0.0001.

Given the contradictory findings reporting on the role of NK cells in EAE in the literature (23, 31, 32), we sought to assess their role in our model and how it is affected by ozanimod treatment. FACS analysis of total circulating and CNS NK cells frequencies showed a marked increase in the mean of peripheral NK cells frequency (37.83%) in mice treated with ozanimod compared to both control (7.60%, P<0.0001) and EAE (12.19%, P=0.0011) mice (**Figure 4A**). Similarly, the mean of CNS NK cells frequency was significantly higher in ozanimod-treated mice (38.69%) compared to control (13.53%, P<0.0001) and diseased untreated (16.09%, P<0.0001) groups (**Figure 4B**).

Upon investigating frequencies of NK cell subsets by assessing differential expression of CD27, no significant changes in the frequencies of both NK subsets (CD27^high^ and CD27^low/-^) were detected in the blood or CNS whether treated or not with ozanimod (**Supplementary Figure 4**). These data indicate that ozanimod treatment affected total NK cells, but not one subpopulation over the other, by increasing frequencies of both subtypes in the circulation and in CNS.

### 3.5 Ozanimod treatment increased the expression of NKG2A on circulating CD27^high^ NK cells and NKG2D on CNS CD27^low/-^ NK cells

**Figure 5:**
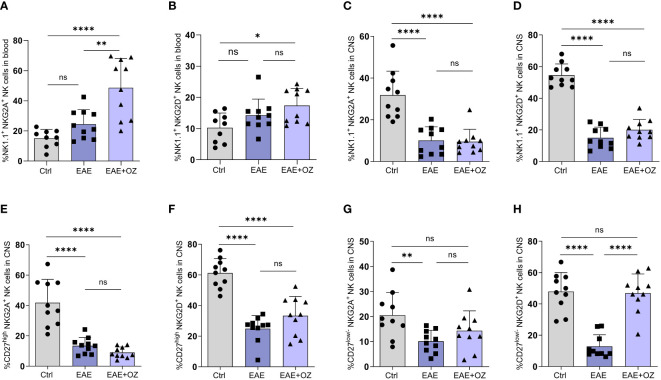
Frequencies of NK cell subsets (CD27^high^ and CD27^low^) and their level of expression of activating (NKG2D) and inhibitory (NKG2A) markers in the circulation and CNS. **(A)** Significant increase in the expression of the inhibitory receptor, NKG2A, was detected compared to **(B)** insignificant change in the expression of the activation receptor, NKG2D, on NK cells in the blood of ozanimod-treated EAE mice compared to those diseased untreated. Insignificant change in the expression of **(C)** NKG2A and **(D)** NKG2D on NK cells in the CNS of EAE mice treated with ozanimod compared to those untreated. Insignificant changes in the level of expression of **(E)** NKG2A, and **(F)** NKG2D on CD27^high^ NK cell subset in the CNS of EAE mice treated with ozanimod compared to untreated mice. **(G)** No detectable changes in the level of NKG2A compared to **(H)** significant increase in the expression of NKG2D on CD27^low/-^ NK cell subset in the CNS of ozanimod-treated EAE mice compared to those untreated. Representative data from two independent experiments are shown (9-10 mice per group). Data are represented as mean ± SD using Mann-Whitney U test. ns P > 0.05, * P ≤ 0.05, ** P ≤ 0.01, **** P ≤ 0.0001.

Assessment of the expression levels of activating (NKG2D) and inhibitory (NKG2A) receptors on NK cells in the circulation and CNS revealed a significant increase in the expression of NKG2A on total circulating NK cells following treatment with ozanimod compared to their levels on NK cells of the control (P<0.0001) and diseased untreated (P=0.006) groups (**Figure 5A**). Treating diseased mice with ozanimod did not alter the level of expression of the activating receptor NKG2D on circulating NK cells (**Figure 5B**). However, ozanimod treatment resulted in a significant increase in the level of expression of the inhibitory receptor NKG2A on circulating CD27^high^ NK cells when compared to diseased but untreated mice (P=0.018) (**Supplementary Figure 5A**). Furthermore, assessment of NKG2D on CD27^high^ NK cells, as well as NKG2D and NKG2A expression on CD27^low/-^ NK cell subsets in the circulation showed no significant changes in response to ozanimod treatment (**Supplementary Figures 5B–D**).

Expression of both receptors on total NK cells in the CNS did not seem to be affected following treatment with ozanimod compared to EAE mice (**Figures 5C, D**). However, while expression of both NKG2D and NKG2A on CD27^high^ NK cell subset (**Figures 5E, F**), as well as the expression of NKG2A on CD27^low/-^ NK cell subset (**Figure 5G**), was not affected by ozanimod treatment in comparison to diseased mice, expression of NKG2D was significantly increased (P <0.0001) (**Figure 5H**). In summary, the present findings suggest that ozanimod has little or no effect on peripheral NK cells activity, but can enhance the activity of NK cells in the CNS by selectively altering the expression of the activating receptor NKG2D on the surface of CD27^low/-^ NK cell subset.

### 3.6 Systemic depletion of NK cells reduced the efficacy of ozanimod in suppressing progression of EAE

**Figure 6:**
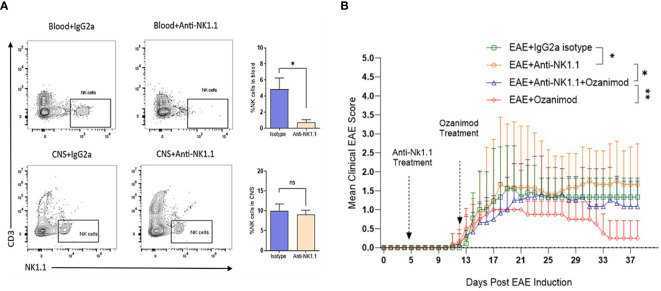
Effect of anti-NK1.1 mAb treatment on the clinical score and body weight of EAE mice. **(A)** Representative flow cytometry profiles of NK cells gated in the blood and CNS of 10-13 weeks old female C57BL/6 mice indicating successful depletion of NK cells in mice treated with anti-NK1.1 mAb in the blood compared to isotype control (85% depletion as represented by the bar graph). Treatment with the depleting antibody did not yield significant depletion of NK cells in the CNS. **(B)** Pre-onset depletion of NK cells using anti-NK1.1 mAb reduced the therapeutic effect of ozanimod as it almost failed to halt progress in the clinical score of EAE mice. Data from two independent experiments are shown (8-12 mice per group). Data are represented as mean ± SD using Mann-Whitney *U* test. ns P > 0.05, * P ≤ 0.05, ** P ≤ 0.01.

The role of NK cells in EAE and in response to treatment with ozanimod was further investigated by depleting NK cells in diseased mice. Successful depletion of circulating NK cells was confirmed by flow cytometry where levels of circulating NK cells in mice treated with anti-NK1.1 mAb showed significant decrease in their percentage (0.72%) compared to control mice (4.84%), which counts for ~85% depletion (**Figure 6A**). Our findings indicate that depleting NK cells at the pre-onset stage [5 days post-induction (dpi)] did not impact the disease onset compared to diseased mice treated with the isotype control, however, it significantly increased the severity of EAE (P=0.018) (**Figure 6B**). Furthermore, anti-NK1.1 mAb treated group exhibited higher clinical scores compared to the group with the combined treatment (ozanimod and anti-NK1.1 mAb) (P=0.011), whereas ozanimod treatment alone resulted in significantly lower clinical scores compared to the combined treatment (P=0.009) (**Figure 6B**). This suggests that NK cells play a significant role in maximizing ozanimod’s effectiveness against EAE.

### 3.7 The efficacy of ozanimod on immune cell frequencies is not affected by NK cell depletion

**Figure 7:**
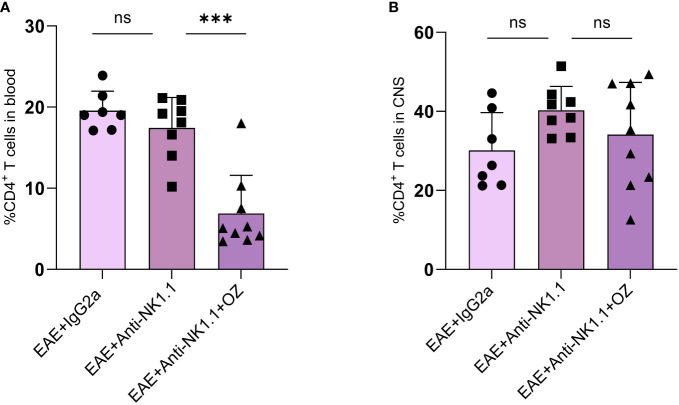
Percentage of CD4^+^ T cells in the blood circulation and CNS of EAE mice following depletion of NK cells using anti-NK1.1 mAb alone or combined with ozanimod treatment. **(A)** Combined treatment of anti-NK1.1 mAb and ozanimod in EAE mice resulted in a significant decrease in the percentage of circulating CD4^+^ T cells but had no effect on the frequency of CD4^+^ T cells in the CNS **(B)**, compared to mice treated with anti-NK1.1 mAb alone. Data from two independent experiments are shown (7-10 mice per group). Data are represented as mean ± SD using Mann-Whitney *U* test. ns P > 0.05, *** P ≤ 0.001.

Impact of NK cells depletion on CD4^+^ and CD8^+^ T cells population in the CNS and the circulation was assessed alone or in combination with ozanimod treatment. Frequencies of circulating CD4^+^ T cells frequencies were markedly decreased in diseased mice depleted of NK cells and treated with ozanimod (6.88%) compared to their untreated counterparts (17.45%) (P=0.001) (**Figure 7A**). However, the depletion of NK cells, whether alone or combined with ozanimod, did not significantly change the frequencies of CD4^+^ T cells in the CNS (**Figure 7B**). Levels of CD8^+^ T cells did not change significantly between NK cell-depleted EAE groups, neither in the blood nor in the CNS (**Supplementary Figure 6**). This suggests that the effect of ozanimod on CD4^+^ and CD8^+^ T cells was not impacted by the depletion of NK cells.

### 3.8 Combination of ozanimod and anti-NK1.1 mAb treatment resulted in a notable increase in the frequency of circulating NK cells in EAE mice

**Figure 8:**
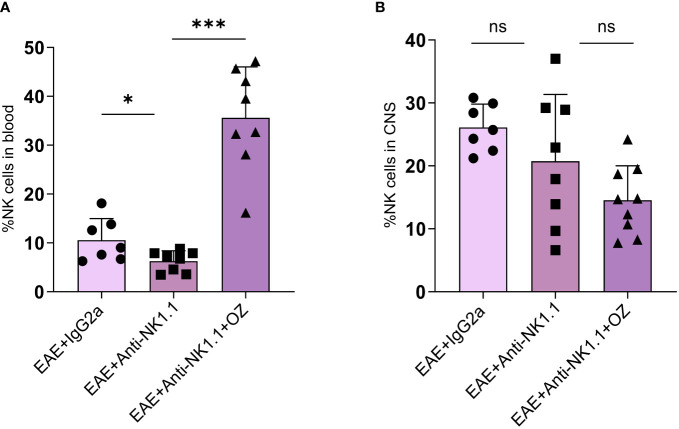
Percentage of NK cells in the blood and CNS of EAE mice treated with anti-NK1.1 mAb alone or combined with ozanimod. **(A)** Treatment with ozanimod following treatment with anti-NK1.1 mAb inhibited the depleting effect of anti-NK1.1 mAb. Combined treatment resulted in a significant increase in the percentage of NK cells in the blood compared to those treated only with anti-NK1.1 mAb **(B)** No significant changes in the percentage of NK cells in the CNS of EAE mice receiving the combined treatment of anti-NK1.1 mAb and ozanimod. Data from two independent experiments are shown (7-10 mice per group). Data are represented as mean ± SD using Mann-Whitney *U* test. ns P > 0.05, * P ≤ 0.05, *** P ≤ 0.001.

Circulating NK cells were successfully depleted in mice treated with anti-NK1.1 mAb compared to the control group (**Figure 8A**). Interestingly, diseased mice depleted of NK cells reported a marked increase in the frequency of NK cells following treatment with ozanimod (35.6%) compared to the untreated group (6.28%) (P=0.0002) (**Figure 8A**). These results may suggest an antagonistic effect of ozanimod on the depleting effect of anti-NK1.1 mAb on circulating NK cells. Notably, frequency of NK cells in the CNS did not significantly change among the different groups (**Figure 8B**). In addition, even though the antagonistic effect of ozanimod treatment on NK cells depletion was evident in the total circulating NK cells, it did not induce any significant changes in the percentage of NK cell subsets whether in the circulation or the CNS (**Supplementary Figure 7**).

### 3.9 CD27^low/-^ NK cell subset enhanced status by ozanimod is affected by the treatment with anti-NK 1.1 antibody

**Figure 9:**
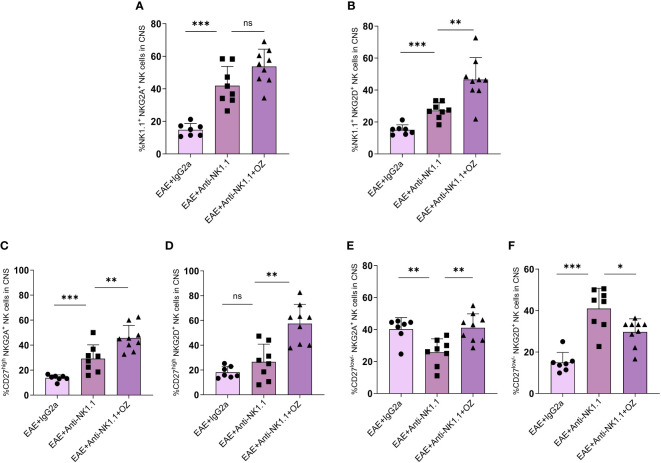
Expression levels of the activating receptor NKG2D and the inhibitory receptor NKG2A on total NK cells and their subsets in the CNS following treatment with anti-NK1.1 mAb alone or combined with ozanimod in EAE mice. **(A)** No detectable effect of the combined treatment of anti-NK1.1 and ozanimod on the expression of NKG2A on NK cells in the CNS compared to **(B)** a significant increase in the expression of NKG2D on NK cells, in comparison to mice treated with anti-NK1.1 mAb alone. Significant increase in the expression of **(C)** NKG2A and **(D)** NKG2D on CD27^high^ NK cell subset in the CNS of mice treated with anti-NK1.1 mAb combined with ozanimod compared to those treated with anti-NK1.1 mAb alone. Significant **(E)** increase of NKG2A and **(F)** reduction of NKG2D expression on CD27^low/-^ NK cell subset in the CNS of EAE mice following combined treatment with anti-NK1.1 mAb and ozanimod compared to mice treated with anti-NK1.1 mAb alone. Data from two independent experiments are shown (7-10 mice per group). Data are represented as mean ± SD using Mann-Whitney U test. ns P > 0.05, * P ≤ 0.05, ** P ≤ 0.01, *** P ≤ 0.001.

The expression of NKG2A and NKG2D on circulating NK cells did not show any significant change between EAE mice treated with anti-NK1.1 mAb and those treated with anti-NK1.1 mAb and ozanimod (**Supplementary Figures 8A, B**). The combined treatment of ozanimod and anti-NK1.1 mAb did not have any extra effect on NKG2A expression on CNS NK cells (**Figure 9A**) but significantly increased NKG2D expression (**Figure 9B**) compared to anti-NK1.1-treated mice. Furthermore, NKG2A and NKG2D expression on circulating CD27^high^ NK cells significantly decreased with anti-NK1.1 treatment compared to the isotype control, remaining unchanged following ozanimod treatment (**Supplementary Figures 8C, D**). Anti-NK1.1 treatment decreased the expression of NKG2A and NKG2D on circulating CD27^low/-^ NK cells compared to the isotype control, and the expression of NKG2D further decreased following the combined treatment (**Supplementary Figures 8E, F**). In contrast, ozanimod treatment increased the frequency of CNS CD27^high^ NK cell subset expressing NKG2A and NKG2D compared to the group treated with anti-NK1.1 mAb (**Figures 9C, D**). NKG2A expression on CD27^low/-^ NK cell subset in the CNS increased significantly in mice that received the combined treatment of anti-NK1.1 mAb and ozanimod compared to those that received anti-NK1.1 mAb only (P=0.002) (**Figure 9E**). Though, significantly reduced percentage of CNS CD27^low/-^ NK cell subset expressing NKG2D was detected in diseased mice with combined treatment compared to the group treated with anti-NK1.1 mAb (P=0.013) (**Figure 9F**). These results indicate that the combined treatment of ozanimod and anti-NK1.1 mAb resulted mainly in an inhibition of the CD27^low/-^ NK cell subset in the CNS, compared to mice treated with anti-NK1.1 mAb alone.

## 4 Discussion

Intensive investigations on the role of NK cells in MS/EAE by other researchers yielded contradictory findings. The focus of our current study was on investigating the effect of ozanimod treatment on disease progression and on NK cells in particular. Following our findings that ozanimod treatment affects NK cells and the expression of their activating and inhibitory receptors, further investigations were conducted to understand whether NK cells contribute to the therapeutic outcome of ozanimod treatment or not. Our findings on the therapeutic potentials of ozanimod on reducing clinical scores of EAE were in line with previously reported findings by Scott and his team (15) using two different doses of ozanimod (0.2mg/kg and 0.6mg/kg). We also showed that treatment with ozanimod was effective in alleviating inflammation and demyelination associated with advanced clinical scores in diseased mice.

Our results were also in agreement with previously reported findings of significantly reduced counts of circulating CD4^+^ and CD8^+^ T cells seen following treatment with ozanimod and other S1PR1 agonists (15, 33). Our results did not reveal any impact of the treatment on the frequencies of CNS-infiltrating activated CD4^+^ T cells (**Figure 3B**). These cells, identified as CCR7^-^ lymphocytes, were previously documented as unaffected by S1PRs modulators (34–36), given the S1P-independent recirculation of activated lymphocytes (CCR7^-^) (12, 37). In addition, we did not look at the subsets of CD4^+^ T cells, which could explain why we did not see any significant change in the frequency of CNS infiltrating CD4^+^ T cells.

With regards to NK cells, our results have shown a significant increase of total NK cells following ozanimod treatment. It is worth mentioning that a similar observation was reported by Schwichtenberg et al. on the immunomodulator closely related to ozanimod, fingolimod (28), where frequencies of circulating NK cells were found to be markedly increased in MS patients treated with fingolimod. However, this was not mirrored by a matching increase in the percentage of either of the NK cell subsets (CD27^high^ and CD27^low/-^). This may be explained by the fact that both immunomodulators are known to interact with S1PR1 and 5 on CCR7-expressing cells but both CCR7 and S1PRs are not co-expressed on NK cell subsets. While CD27^low/-^ NK cell subset expresses high levels of S1PR-5 and low levels of CCR7 (27), CD27^high^ NK cell subset expresses high levels of CCR7 and low levels of S1PR-5 (38, 39). Moreover, no changes were detected in the frequencies of circulating NK cells expressing NKG2D in diseased and ozanimod-treated mice, but a significant increase in the percentage of NK cells, particularly the CD27^high^ NK cell subset, expressing NKG2A was observed, suggesting exhaustion status or suppression of circulating NK cells (**Figures 5A, B**; **Supplementary Figure 5A**). However, treatment with ozanimod particularly activated the CD27^low/-^ NK cell subset in the CNS (**Figure 5H**). Nevertheless, combining ozanimod with anti-NK1.1 mAb resulted in a reversal of the expression of NKG2D receptor on this subset (**Figure 9F**). This reversal appears to be linked to the therapeutic potential of ozanimod, as indicated by our findings.

Following to the initially observed elevation in the frequency of NK cells in diseased mice, we investigated whether that increase plays a role towards the detected therapeutic outcome of ozanimod treatment. The findings from our study on depleting NK cells indicate that the efficacy of ozanimod treatment is reduced when combined with NK cells depleting antibody. Our findings confirmed a regulatory role of NK cells since their depletion yielded higher clinical scores (**Figure 6B**). This is consistent with the findings by others, where depletion of NK cells resulted in severe progression of EAE (29, 39, 40). In addition, our results showed that ozanimod alone improved the clinical score more efficiently than when it is combined with anti-NK1.1 mAb in EAE mice, suggesting that NK cells play a role in the ozanimod induced remission of EAE (**Figure 6B**).

Our findings also revealed that even though frequency of CNS NK cells did not change following peripheral NK cell depletion (**Figure 8B**), their activity status was altered as shown by increased expression of NKG2D on total NK cells following the combined treatment of anti-NK1.1 mAb and ozanimod. However, this increase was confirmed in the CD27^high^ NK cell subset, whereas the CD27^low/-^ NK cell subset exhibited lower levels (**Figure 9**). It is noteworthy that the severity of EAE was inversely proportional to the level of expression of NKG2D on CNS CD27^low/-^ NK cell subset supporting the suggestion that upregulation of this receptor on CNS CD27^low/-^ NK cell subset is crucial for improving disease outcome and maybe delaying the relapses in EAE mice. It is important to note that with the limited number of studies investigating the effect of ozanimod treatment in EAE, our findings serve to support the therapeutic potentials of the drug and report the novel finding of the role of NK cells in impacting the treatment. Though, future studies are needed to validate the findings and study the mechanism by which the two treatments counteract each other’s effect. Furthermore, exploring the mechanism by which ozanimod impacts the activation and inhibition receptors of NK cells would be intriguing.

In conclusion, the immunomodulatory drug ozanimod demonstrated significant disease ameliorating potentials in EAE. This therapeutic effect is believed to be associated with enhanced expression of NKG2D on CD27^low/-^ NK cell subset in the CNS. Further, results from the NK cell depletion study confirm the crucial role of NK cells in boosting the efficacy of ozanimod treatment in reducing the severity of EAE.

## Data availability statement

The raw data supporting the conclusions of this article will be made available by the authors, without undue reservation.

## Ethics statement

The animal study was approved by Ethical approval committee, College of Medicine and Health Sciences, United Arab Emirates University. The study was conducted in accordance with the local legislation and institutional requirements.

## Author contributions

AK-S and MS conceived and together with MH supervised the study. DK, MH, and AN performed the experiments. DK, MH, MS, AT, and ZR analyzed the data. DK and MH wrote the first draft of the manuscript. All authors contributed to the article and approved the submitted version.
