# Supplementary material for: Ozanimod-mediated remission in experimental autoimmune encephalomyelitis is associated with enhanced activity of CNS CD27low/- NK cell subset
Source: Front Immunol. 2024 Mar 12;15:1230735. doi: 10.3389/fimmu.2024.1230735 (PMC10963535; doi:10.3389/fimmu.2024.1230735)
Supplement: Supplementary file 1 [file DataSheet_1.docx]

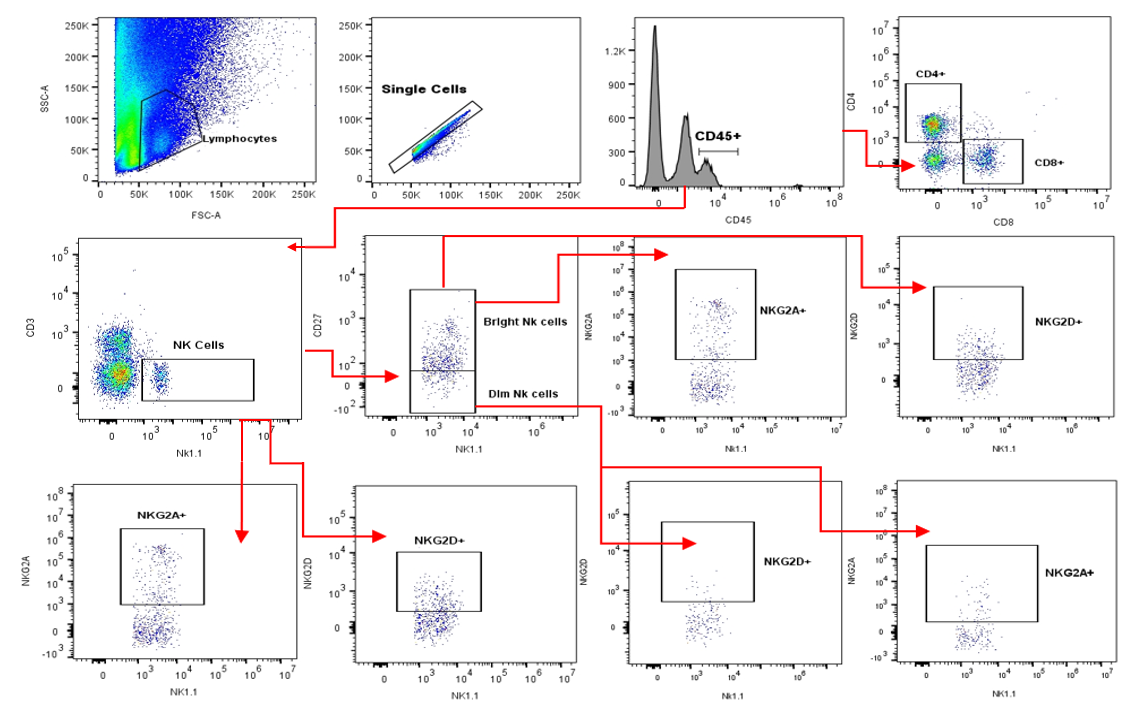
**Supplementary Figures**

Supplementary Figure 1: Representative flow cytometry dot plots of the applied gating strategy for CD4^+^, CD8^+^ T, and NK cells, as well as expression of NKG2A and NKG2D on NK cells in the CNS. Blood was also gated using the same strategy. Cells of interest were selected based on size and granularity in the forward scatter (FSC-A) and side scatter (SSC-A). Doublets were then excluded in FSC-A/ /FSC-H, and CD45^+^ cells were all selected. CD45^high^ lymphocytes were then split into CD4^+^ and CD8^+^ T cells. NK cells were identified as CD3^-^ NK1.1^+^ and NK cell subsets were distinguished according to the differential expression of CD27 marker. CD27^high^ NK cell subset was gated as NK1.1^+^ CD27^high^ while CD27^low/-^ NK cell subset was gated as NK1.1^+^ CD27^low/-^. Expression of activating (NKG2D) and inhibitory (NKG2A) receptors was determined on total NK cells and their subsets CD27^high^ and CD27^low/-^.

Supplementary Figure 2: Representative clinical scores of individual mice. Data are shown as mean ± SD using Mann-Whitney *U* test. ns P > 0.05, ** P ≤ 0.01, **** P≤ 0.0001.


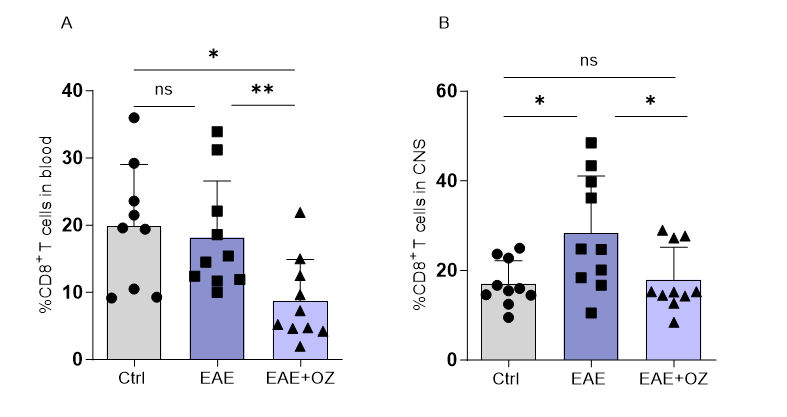


Supplementary Figure 3: Percentages of CD8^+^ T cells in the (A) blood and (B) CNS of EAE mice were decreased following treatment with ozanimod. Representative data of two independent experiments (9-10 mice per group) are shown as mean ± SD using Mann-Whitney *U* test. ns P > 0.05, * P ≤ 0.05, ** P ≤ 0.01.


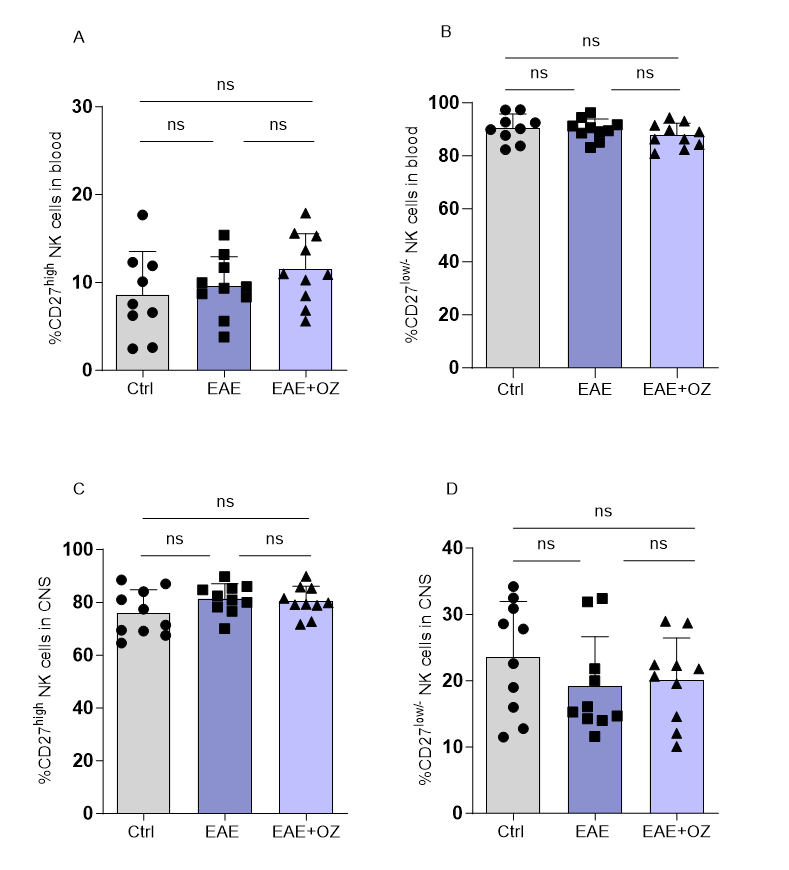


Supplementary Figure 4: Percentages of CD27^high^ and CD27^low/-^ NK cell subsets in the (A, B) blood and (C, D) CNS of EAE mice were not affected following treatment with ozanimod. Representative data of two independent experiments (9-10 mice per group) are shown as mean ± SD using Mann-Whitney *U* test. ns P > 0.05.


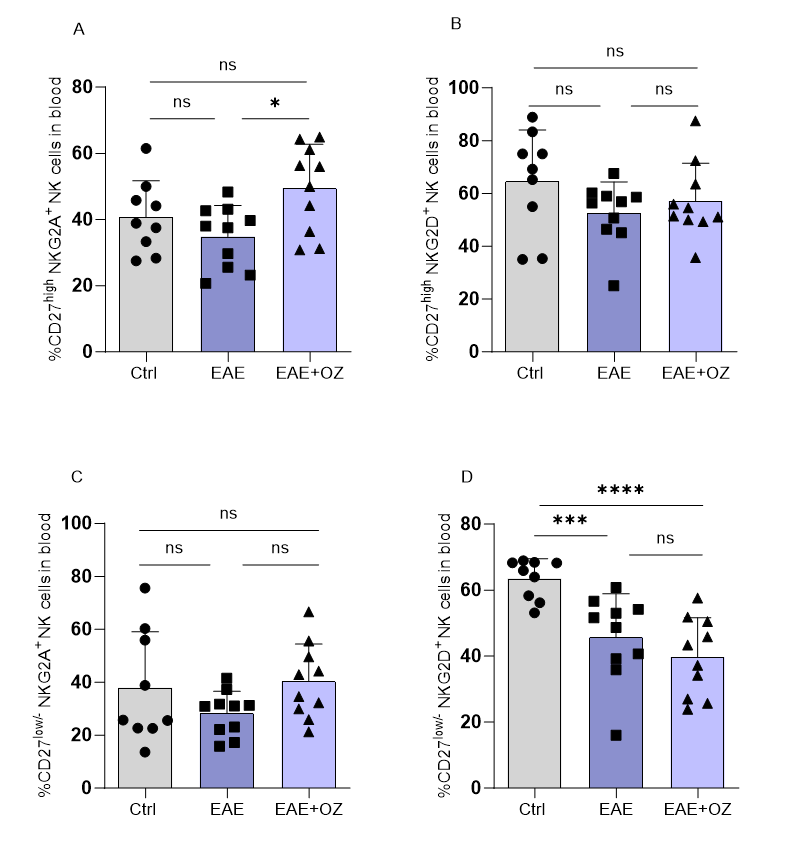


Supplementary Figure 5: (A) Significant increase in the expression of NKG2A on circulating CD27^high^ NK cell subset in EAE mice treated with ozanimod. (B) Unaltered expression of NKG2D on circulating CD27^high^ NK cell subset in EAE mice treated with ozanimod. Insignificant changes in the expression of (C) NKG2A and (D) NKG2D on CD27^low/-^ NK cell subset in the CNS of EAE mice treated with ozanimod compared to untreated diseased mice. Representative data of two independent experiments (9-10 mice per group) are shown as mean ± SD using Mann-Whitney *U* test. ns P > 0.05, * P ≤ 0.05, *** P ≤ 0.001, **** P≤ 0.0001.


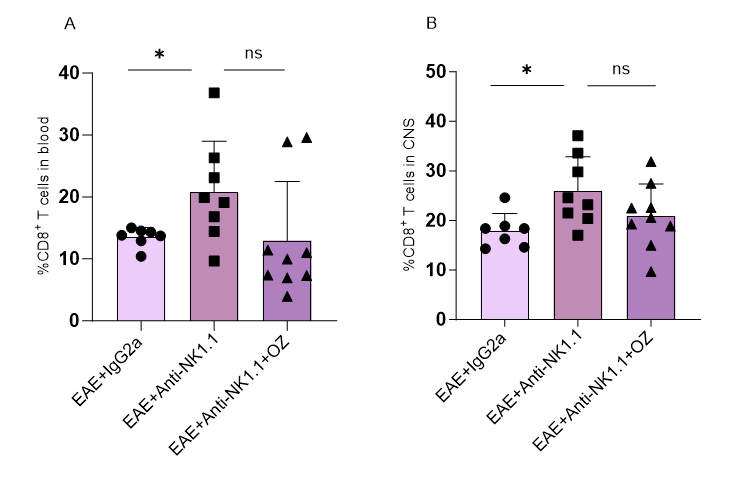


Supplementary Figure 6: Percentages of CD8^+^ T cells in the (A) blood and (B) CNS of EAE mice were not affected following the combined treatment of anti-NK1.1 mAb and ozanimod compared to anti-NK1.1 mAb alone or isotype control-treated groups. Representative data of two independent experiments (7-10 mice per group) are shown as mean ± SD using Mann-Whitney *U* test. ns P > 0.05, * P ≤ 0.05.


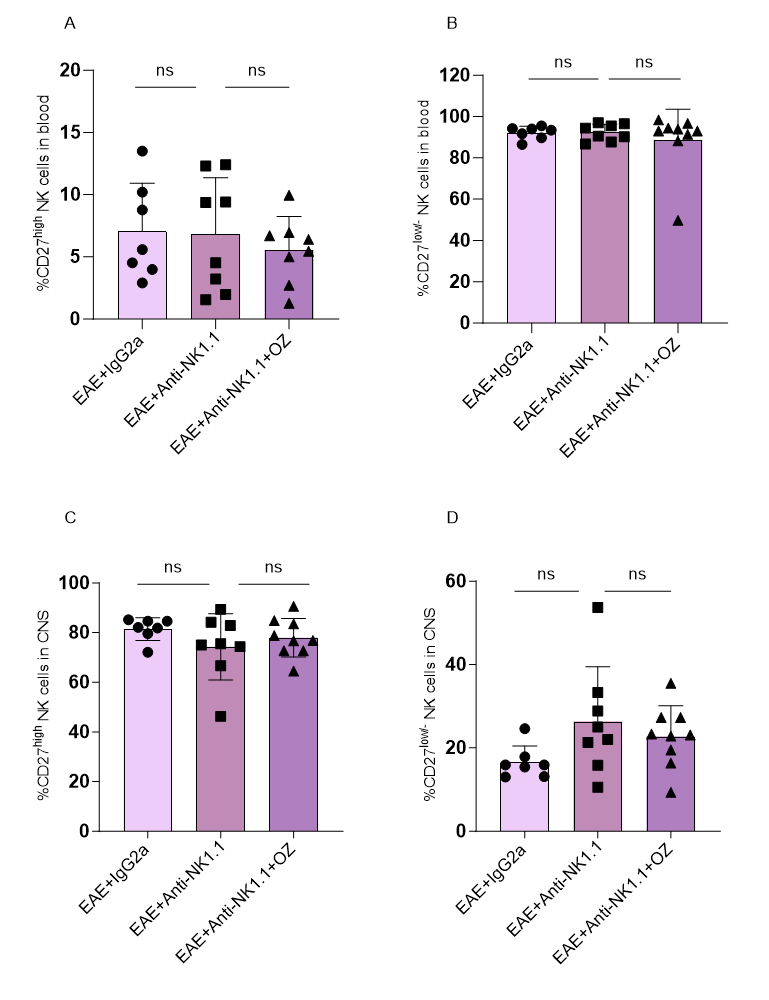


Supplementary Figure 7: Percentages of CD27^high^ and CD27^low/-^ NK cell subsets in the (A, B) blood and (C, D) CNS of EAE mice were not affected following the combined treatment of anti-NK1.1 mAb and ozanimod. Representative data of one single experiment (7-10 mice per group) are shown as mean ± SD using Mann-Whitney *U* test. ns P > 0.05.


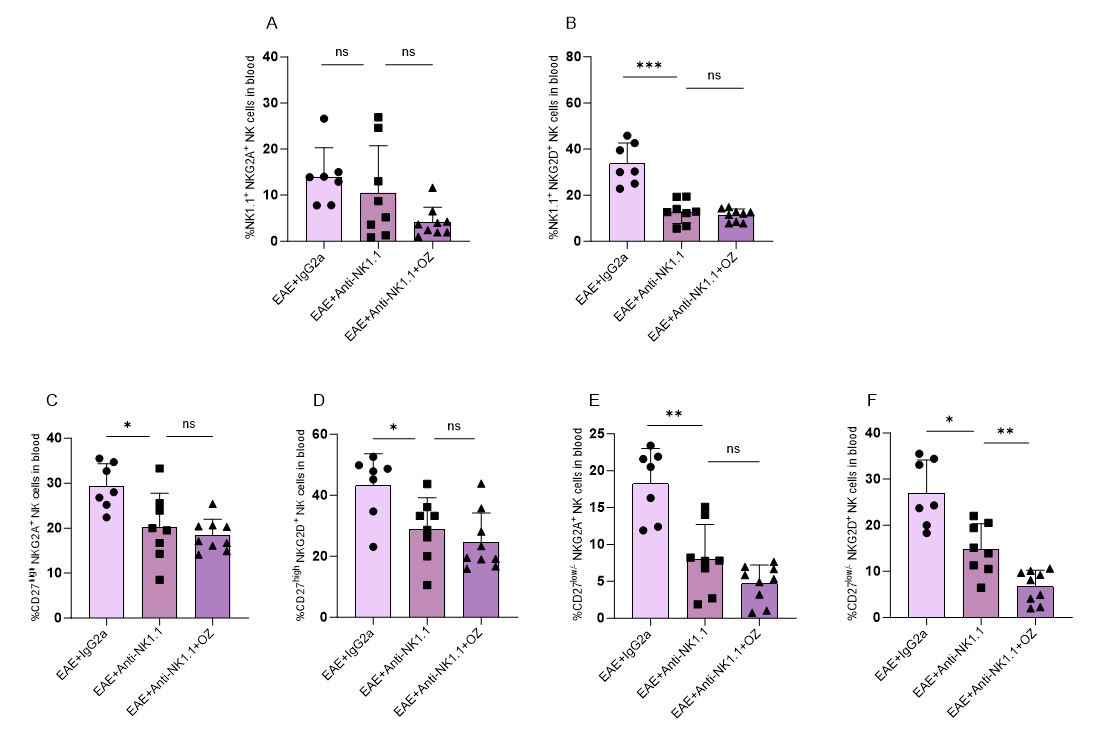


Supplementary Figure 8: Insignificant changes in the expression of (A) NKG2A and (B) NKG2D on NK cells in the blood of EAE mice following the combined treatment of anti-NK1.1 mAb and ozanimod. This combined treatment did not change the expression of (C) NKG2A and (D) NKG2D on CD27^high^ NK cell subsets or of (E) NKG2A in CD27^low/-^ NK cell subsets in the blood. Decreased expression of (F) NKG2D on CD27^low/-^ NK cell subsets following the combined treatment of anti-NK1.1 and ozanimod. Representative data of two independent experiments (7-10 mice per group) are shown as mean ± SD using Mann-Whitney *U* test. ns P > 0.05, * P ≤ 0.05, ** P ≤ 0.01, *** P ≤ 0.001.
